# Gynecologists and Obstetricians Working Group to Face the COVID-19 Pandemic in Brazil: Successful Experience to be Followed

**DOI:** 10.1055/s-0041-1736170

**Published:** 2021-09-21

**Authors:** Silvana Maria Quintana, Geraldo Duarte

**Affiliations:** 1 Faculdade de Medicina, Universidade de São Paulo, Ribeirão Preto, SP, Brazil

In addition to causing relevant changes in the global routine, the COVID-19 pandemic status announced and recognized by the World Health Organization (WHO) on March 11, 2020, made it clear that no country was ready to face an infectious disease that spread rapidly and involved objective risk of death. The retrospective assessment of the sequence of events and outcomes over the past 17 months leads us to reflect on what was done, what could have been done, or what should have been done differently. One of the great challenges in this context was defining which essential health services should not undergo continuity solutions. With regard to women's health, part of the care routine was interrupted due to the emergency situation caused by COVID-19, while others continued to develop their activities following the health safety protocols. Reproductive planning services, care to victims of domestic and sexual violence and oncology stand out among those suffering significant reduction in the offer of services for diagnosis and treatment, which will certainly bring future and important repercussions for the health of the population.

In relation to obstetric care specifically, women continued to become pregnant and have their pregnancies resolved. At the beginning of the pandemic, we feared that women in the pregnancy-puerperal cycle would acquire SARS CoV-2 and based on the experience with the influenza epidemic in 2009, would present unfavorable outcomes.

In an environment of insecurity, personal opinions and prescription of treatments without scientific evidence, COVID-19 promoted an unprecedented public health crisis that required the development of strategic plans contextualizing clear communication about the disease and its particularities and judicious allocation of resources. Services for the care of patients infected with SARS CoV-2 had to be organized to ensure the safety of patients, health professionals and ancillary staff, including cleaning and administrative services, among others. In obstetric units, the flow of patients had to be organized, defining which units would be used to care for patients with COVID-19. Teams had to be prepared and a strategy integrating the units and teams from different areas and hospitals was established to allow the synergy of teams for the proper care provision in a structure with a workforce at maximum capacity.

Although different obstetric care services have been organized in our country and protocols were developed in accordance with international quality standards, COVID-19 continued to spread, confirming that the disease had a worse evolution among women in the pregnancy-puerperal cycle. Over time, it was learned that there was greater demand and need for hospitalization, care in the Intensive Care Unit (ICU), invasive ventilation and even death, compared with non-pregnant women.

In view of this, the Ministry of Health of Brazil constituted a working group composed of 17 specialists in the area of women's health from different university institutions to develop strategies aimed at fighting the pandemic for this population segment, having as background the reduction of maternal mortality by COVID-19. It was the perfect partnership between the Ministry of Health and academia! The first challenge overcome by this group was to standardize care for pregnant and puerperal women with suspicion or diagnosis of this viral infection, addressing the diagnosis, treatment, attention to antenatal care, ICU care and, obviously, a care flow model for the health services attending pregnant and puerperal women affected by this infection. Such a strategy should be feasible and accessible for adoption in a continental country, with notable differences in the health and care conditions of its population. Based on the available scientific evidence, since there was no randomized clinical trial in pregnant women, and on the expertise of the group that met virtually to discuss the topic each week, a manual 1 was created.

The second challenge was to expand the information contained in the manual to reach all services, from north to the south of Brazil and help their adaptation to the new guidelines, leaving their realities and routines and adapting to the care of women with a disease hitherto unknown in our country. Real clinical cases started to be discussed with the different obstetric care services throughout Brazil. 2 The contact with health teams from all states, with exchange of experiences and learning was relevant to the progress of work. There were ∼160 virtual meetings, twice a day, five days a week, in all Brazilian states. There was excellent receptivity from local teams, who shared their difficulties and adopted the recommendations of the working group.

With case discussions across the country, the working group realized the need for an effective participation of public universities during the challenge of a health crisis such as the one caused by the COVID-19 pandemic. Additionally, the group sought to ensure easier access to information, so that professionals could watch educational videos during their shifts, according to their needs. In partnership with the Pan American Health Organization (PAHO), short classes 3 with the main topics of the manual were recorded, creating an easily accessible video library that could be watched anywhere and, given its practical character, assist in care.

The manual was updated as knowledge about the disease evolved and the working group deepened, particularly in studies on the benefits and risks of vaccination for COVID-19 in pregnant and puerperal women, as maternal mortality due to COVID -19 in Brazil is one of the highest in the world. Thus, based on the available scientific literature, the working group became convinced that vaccines against COVID-19 could help to change the reality of such an unfavorable outcome. Data from the Brazilian Obstetric Observatory COVID-19 (OOBr COVID-19). https://observatorioobstetrico.shinyapps.io/covid_gesta_puerp_br/ ), updated with data made available in the Information System for the Epidemiological Surveillance of Influenza (Portuguese acronym: SIVEP-Influenza) of the Ministry Health, became a valuable resource to be part of the reality of the working group. The OOBr COVID-19 is part of the Brazilian Obstetric Observatory project, which provides an interactive and dynamic monitoring platform, scientifically based public data analysis and the dissemination of quality and relevant information in the area of Maternal and Child Health. Its data showed us the number of pregnant and puerperal women with Severe Acute Respiratory Syndrome (SARS), the weekly number of deaths in the country and the highest risk gestational trimester. These data allowed the working group to reach a definitive conclusion on the urgent use of the vaccine in pregnant and puerperal women with or without comorbidities, who should receive the vaccine against COVID-19 at any stage of pregnancy. In this context, the partnership with support of the Gynecology and Obstetrics Specialty Societies in the country was essential to broaden the debate and publicize the safety and effectiveness of vaccines during pregnancy and puerperal period. The Brazilian Federation of Gynecology and Obstetrics Societies - FEBRASGO constituted a Temporary Specialized National Commission of COVID (Portuguese acronym: CNET COVID), confirming the lead role of the working group in the national scenario.

As a result of all this work, the vaccination of pregnant and puerperal women was included in the National Immunization Plan of the Ministry of Health (Portuguese acronym: PNI-MS) and the results shown in the OOBr Covid-19 demonstrate that the vaccination of these women was the right decision. Today, we are facing a situation of more hope for pregnant and puerperal women in the face of the pandemic, as the effects of vaccination reflected in the reduction of maternal mortality by ∼92% in our country.

An interesting aspect during the discussions of clinical cases with the different services was that many difficulties for the planned changes were beyond the decision-making power of health teams, that is, the material needs and changes in procedures were under the responsibility of local or regional health managers. With the help of the Oswaldo Cruz Foundation (FIOCRUZ), an institution linked to the Ministry of Health, meetings of the working group with state managers were held, pointing out the difficulties observed in the discussions and suggesting changes. The lack of harmony and communication difficulties in the health network in our country drew attention.

The experience acquired in this working group for a common good, facing a pandemic, showed that the performance of this group can be a model to be applied to combat maternal mortality resulting from different causes in our country. In view of this scenario, our assessment of coping with COVID-19 in Brazil, particularly considering pregnant and puerperal women, is extremely favorable. Within the limitations inherent in a developing nation, it was very positive to observe that our response was as adequate as those observed in countries with more favorable socioeconomic conditions.

Together - Educational Institutions, Ministry of Health, research and the health care network - we are stronger to overcome the difficulties that Brazil is facing and will still face. Brazilian pregnant women deserve this care!
